# Formation and Penetration Properties of a Shaped Charge with Zr_41.2_Ti_13.8_Cu_12.5_Ni_10_Be_22.5_ Liner

**DOI:** 10.3390/nano12223947

**Published:** 2022-11-09

**Authors:** Xudong Zu, Taian Chen, Yaping Tan, Hao Chen, Zhengxiang Huang

**Affiliations:** School of Mechanical Engineering, Nanjing University of Science and Technology, 200 Xiao Ling Wei Street, Nanjing 210094, China

**Keywords:** Zr_41.2_Ti_13.8_Cu_12.5_Ni_10_Be_22.5_, amorphous alloy, high-speed particle jet, shaped charge

## Abstract

In the military field, determining how to increase the hole-expanding ability of shaped charge warheads is a key and difficult issue with respect to warhead development. Amorphous alloys have grains or grain boundaries, with unique mechanical properties. Zr_41.2_Ti_13.8_Cu_12.5_Ni_10_Be_22.5_ can be used as the liner material of shaped charges, resulting in high-speed particle flows that differ from those of traditionally shaped charges. In this paper, based on the analysis of the mechanical response characteristics of Zr_41.2_Ti_13.8_Cu_12.5_Ni_10_Be_22.5_ and its fracture morphology under impact, combined with the formation theory of shaped charge jets, a semi-empirical formula is derived to calculate the velocity of non-cohesive high-speed particle flow considering the elastic strain energy loss. Additionally, the reliability of the proposed theoretical model is verified through experiments. The penetration process of Zr-based amorphous alloy high-speed particle flow into a concrete target is theoretically analyzed, and the penetration stages of the high-speed particle flow into the target are clearly distinguished. Combined with the penetration theory of shaped charge particle jets, a high-speed particle flow penetration model is proposed, and a pore expansion model is established through an energy method. The experimentally obtained data on depth of penetration are in agreement with the theoretical calculation results.

## 1. Introduction

To attack a target underground, a tandem warhead with a shaped charge as the front charge is typically used. When a shaped charge is used as the forward charge in a tandem warhead to penetrate rock and soil, a large hole diameter of the penetration is vital. The hole diameter of penetration of the front charge has a significant effect on the penetration ability of the follow-through charge. Furthermore, the hole diameter of a normally shaped charge should be no more than 0.6 times the charge diameter (CD). When the diameter of the penetration hole is more than 0.7 CD, the depth of penetration (DOP) of the follow-through charge is increased significantly.

Amorphous alloys are a class of crystalline metals produced by avoiding crystallization during the solidification process of metals. They have high-strength properties, high toughness, and good process formability. The strengths of Zr-based amorphous materials show a trend of first decreasing and then increasing with increased strain rate; when the strain rate is about 3000 s^−1^, the shear deformation mechanism of Zr-based amorphous materials changes, and the deformation mode is similar to that of Zr-based crystalline materials. These materials exhibit the strain-hardening mechanism of crystalline materials. Moreover, with increased strain rate, the nanocrystal content gradually increases, strain hardening intensifies, and the compressive strength increases. Furthermore, a Zr-based amorphous alloy material can be used as the liner material. Consequently, the liner can form a high-speed particle flow driven by the explosive. When penetrating reinforced concrete, the high-speed particle jet can form a large penetration hole (0.7 times the diameter of the charge), enabling continuous penetration of the warhead with the use of a rear warhead.

Amorphous alloy materials have good mechanical and physical properties, such as high strength, hardness, and bending strength, as well as good wear resistance, fracture toughness, and corrosion resistance. This is because their microstructures show short-range order, long-range disorder, and no defects such as dislocations and vacancies; they are metastable structures. These materials have been extensively studied in the past few decades. However, almost all amorphous alloy materials are considered brittle materials at temperatures in the range of 23 °C–25 °C, owing to the local deformation caused by the infinite expansion of their single shear bands, which severely limits their application as structural materials. Therefore, before conducting engineering application research on bulk Zr-based amorphous alloy materials, mechanical property analysis should be carried out.

Bruck [1] and other scholars found that the mechanical properties of Zr_41.2_Ti_13.8_Ni_10_Cu_20_Be_22.5_ bulk amorphous alloy are not sensitive to the strain rate. Lu [2] confirmed this conclusion. However, when in the cold liquid phase, Zr_41.2_Ti1_3.8_Ni_10_Cu_20_Be_22.5_ also exhibits strain-rate-sensitive mechanical properties. The bulk amorphous alloys Zr_57_Ti_5_Ni_8_Cu_20_Al_10_ [3] and Pd_40_Ni_40_P_20_ [4] have been found to have a negative effect on the mechanical properties of strain rate; that is, increased strain rate, the strength of the material increases. In contrast, the strain rate of bulk amorphous alloys Nd_60_Fe_20_Co_10_Al_10_ [5], Zr_25_Ti_40_Ni_8_Cu_9_Be_18_ [6], and Zr_16_Ti_45_Ni_9_Cu_10_Be_20_ [7] has a positive effect on their mechanical properties. Therefore, owing to the influence of various factors, such as the composition of the amorphous alloy, a general conclusion often cannot be drawn about the strain rate effect; therefore, the relevant mechanical properties of the amorphous alloy used in the study should be investigated. The three-dimensional, finite-deformation-based constitutive equations for metallic glasses developed by Thamburaja and Ekambaram [8] have been proven capable of accurately recording the deformation behavior of amorphous alloys, such as Pd-based amorphous alloy and La61:4Al15:9Ni11:35Cu11:35 bulk metallic glass.

In the context of the application of amorphous material liners, Zheng [9] proposed W-Cu-Zr non-metallic liner materials. Based on research on the comprehensive mechanical properties of this material, it was assumed that its use as liner material was conducive to improving the penetration power of warheads. Walters and Kecskes [10] studied the jet formation and penetration of metal-glass shaped charge liners, demonstrating that the jet mainly presented a high-speed particle flow pattern composed of a large number of particles. The movements of high-speed particles were independent of each other, and the particles had certain radial velocities. When the blasting height was 2.3 times the diameter of the charge, an opening effect 0.51 times the diameter of the charge could be achieved. However, no detailed studies have been conducted on the specific formation process, the formation mechanism of the jet formed by the amorphous alloy hood, or the penetration model of the high-speed technical particle flow.

In this study, Zr_41.2_Ti_13.8_Cu_12.5_Ni_10_Be_22.5_ amorphous alloy material was taken as the research object. First, the dynamic mechanical properties of the material and the damage characteristics of the material under impact were analyzed. The formation process and characteristics of high-speed particle flow were analyzed in depth under explosive driving when the Zr_41.2_Ti_13.8_Cu_12.5_Ni_10_Be_22.5_ amorphous alloy material was used as the liner material. The results reported herein can provide technical guidance with respect to the selection of new materials suitable for shaped charge liners of shaped charge assault warheads, as well as technical support for the design of high-speed particle jet warheads.

## 2. Experimental Study

### 2.1. Preparation of Zr_41.2_Ti_13.8_Cu_12.5_Ni_10_Be_22.5_

Before the engineering application of Zr-based amorphous alloy materials, mechanical property analysis should be carried out. Moreover, because the fabrication process of an amorphous alloy influences its mechanical properties, all the samples investigated in the present study were prepared by the copper mold casting method.

**Figure 1:**
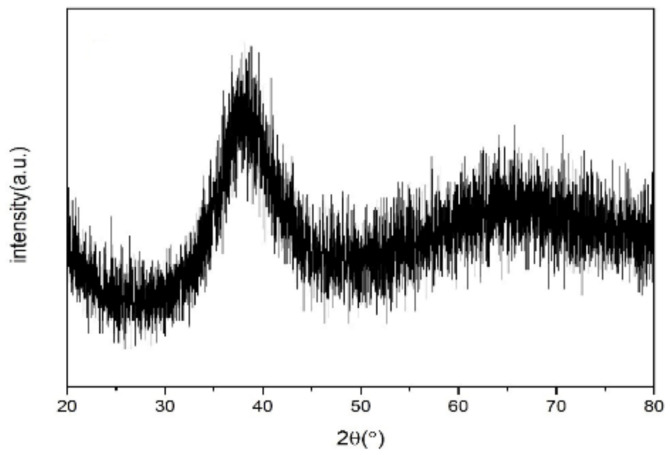
X-ray diffraction spectra of Zr-based amorphous alloys.

**Figure 2:**
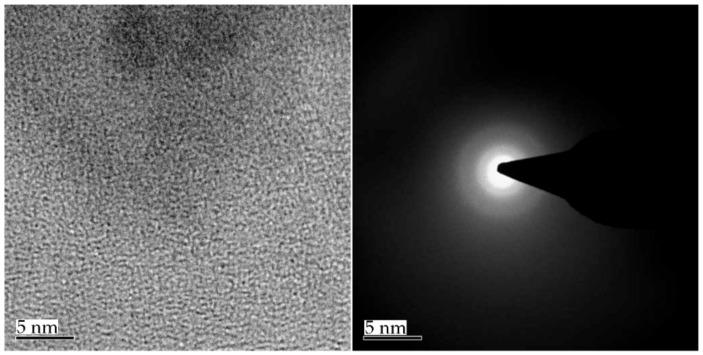
HRTEM image and diffraction spot of Zr-based amorphous crystals.

**Figure 3:**
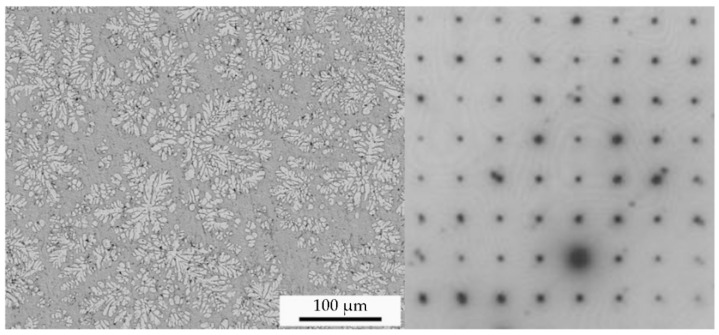
Microstructure and diffraction pattern of Zr-based amorphous material.

Various metal materials with purities of more than 99.5% were selected as raw materials. After ultrasonic cleaning, the raw materials were put into a water-cooled copper crucible according to specified proportions, and an arc melting furnace was used to prepare ingots of Zr-based amorphous alloys under the protection of high-purity argon gas. Repeated smelting was applied more than three times to ensure the uniformity of the alloy composition. The samples were prepared by spray casting in a vacuum induction furnace and processed to the sizes of the test samples. After the sample was polished with sandpaper and the polishing solution was fully polished to a surface roughness of about 0.2 μm, the surface of the sample was observed by scanning electron microscopy (SEM) to ensure that there were no defects that would affect the test results. Figure 1 shows the X-ray diffraction (XRD) spectrum of the Zr-based amorphous alloy. The curve had only one broad diffraction peak, and no crystal phase diffraction peak was evident, indicating that the sample was successfully prepared. Through SEM, transmission electron microscopy (TEM), high-resolution TEM (HRTEM), and other tests, the structures of the amorphous material and its composite materials were confirmed. Figure 2 shows the HRTEM results and XRD diffraction spot of the Zr-based amorphous material. Figure 3 shows the microstructure and diffraction pattern of the Zr-based amorphous material.

### 2.2. Mechanical Properties

#### 2.2.1. Mechanical Properties Test

Bulk metallic glass (BMG) was measured using a universal testing machine and a split Hopkinson pressure bar (SHPB). To investigate the fracture mechanisms, the fracture surfaces of the specimens were observed by SEM after testing.

Specimens with diameters of 3 mm and heights of 6 mm were prepared for quasi-static compressive tests, and specimens with diameters of 5 mm and heights of 3 mm were prepared for dynamic compression tests. The specimens were sanded with 1000-, 2000-, and 3000-grit sandpaper, then fully polished with a polishing liquid with a particle size of 0.3 μm for 10 min. The surface of the specimen was observed by SEM, and no flaws were found. The laser scanning confocal microscope detection results showed that the surface roughness values of all the specimens were about 0.2 μm. The surface states of all the specimens were similar. Thus, the experimental results were not affected by the surface states of the specimens.

**Figure 4:**
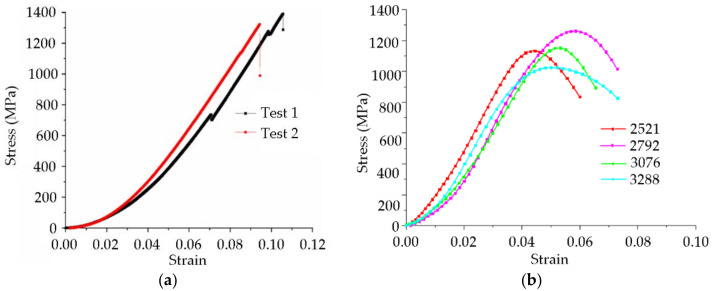
Stress–strain relations of specimens at different strain rates: (**a**) quasi-static compressive tests and (**b**) dynamic compressive tests.

Figure 4 shows the stress–strain relations of the Zr_41.2_Ti_13.8_Cu_12.5_Ni_10_Be_22.5_ material from quasi-static and dynamic compressive tests at room temperature. The curves were displaced by a 2% strain so that multiple curves were clearly visible. In contrast to conventional metals, the Zr-based amorphous alloy specimens exhibited a sudden drop in stress at the end of the linear elastic stage and the beginning of plastic deformation. No macroscopic plastic deformation, strain hardening phenomenon, or brittle behavior were evident. When the samples were compressed, they first underwent elastic deformation, the stress reached the dynamic yield strength of the material, and the material broke immediately. The relevant data are shown in Table 1.

When a high strain rate was applied to the Zr-based amorphous alloy specimens, they exhibited a brittle fracture mechanism similar to that observed in the static test. Under low strains, the stress–strain curves basically followed Hooke’s law, and the materials could be regarded as being in an elastic stage until the stress reached the dynamic yield strength of the material. After the stress reached the yield strength, the specimens broke immediately, and the stress–strain curves decreased rapidly. The dynamic plastic deformation was almost zero. Based on the quasi-static compression tests, the Zr-based amorphous alloy could be regarded as a brittle material.

A comparison of the experimental data under quasi-static conditions showed that the compression elastic strain limit of the Zr-based amorphous alloy of this composition under different dynamic strain rates at room temperature was less than that under static conditions. However, the elastic modulus under room-temperature dynamic conditions was higher than that under static conditions. With increased strain rate, the elastic modulus was basically unchanged, with a slight decrease because the static compression test had a low loading rate, and the shear band was able to develop and mature, causing a large amount of elastic deformation to accumulate. In the dynamic compression test, the load loading rate was fast, and the shear band promoted cracking immediately after it was generated. Thus, the accumulated elastic strain was low. With increased strain rate, this trend still occurred, but the numerical change was minimal. As a result, the static compressive yield strength of the amorphous alloy was greater than the dynamic compressive yield strength.

In addition, the yield strengths of the Zr-based amorphous alloy specimens showed trends of increasing first and then decreasing with increased strain rate; that is, the materials first exhibited strain rate hardening and then strain rate softening, and the inflection points occurred at a strain rate of approximately 3000 s^−1^. This may have been caused by the excessive heat accumulation inside the material after the critical strain rate was exceeded, and the thermal softening effect was evident. The strain-hardening phenomenon of the specimen was not evident during the whole room-temperature dynamic compression process. After the sample reached the yield point, it did not break instantaneously as in a quasi-static test, but there was a small plastic yield stage. Because there were no defects, such as grain boundaries and dislocations in the amorphous alloy structure, the plastic yield can be explained by the cluster model, in which a shockwave forms an uneven stress field in and around the cluster. Under the influence of the free volume, plastic rheology occurred locally in the sample. When the shockwave pressure was high enough, the free volume broke, released, combined, further matured from the cluster, and finally formed a microshear band along the shear stress direction. Distinct “sparks” occurred during the test. After the SHPB test, a small number of residual sample fragments appeared on the tungsten carbide gasket at the end of the compression rod because during the dynamic test, the impact load was applied for a very short time, the specimens rapidly deformed, and the considerable elastic energy stored in the deformation process was released in a short time. This led to a rapid temperature rise of the specimens, resulting in some specimen fragments melting and adhering to the tungsten carbide shims. Table 2 shows the test data for the Zr-based amorphous alloy.

#### 2.2.2. Dynamic Constitutive Equations of Zr-Based Amorphous Alloy

Microvoids and microcracks are prevalent in amorphous alloys, reducing the strengths of these materials and inducing the accumulation of material damage. The JH2 constitutive model can comprehensively account for the influences of the strength, damage, and pressure and is primarily used to describe the dynamic mechanical responses of brittle materials in an environment with a high strain rate, high pressure, large deformation, and material damage and failure. JH2 can reflect the transition of amorphous alloys from a complete state to a fractured state. Owing to the elastic brittle and non-porous material properties of the materials investigated in this study, the JH2 constitutive model was selected to describe the dynamic mechanical behavior of the Zr-based amorphous alloy.

The material density of the Zr-based amorphous alloy was 6.11 g/cm^3^, the elastic modulus was 97 GPa, the Poisson’s ratio was 0.35, the Hugoniot elastic limit was *σ_HEL_* = 6.15 GPa, the spalling fracture strength was *σ_s_* = 2.35 GPa, the fracture strength was *σ_F_* = 1.8 GPa, and the Green’s constant was *γ* = 1.85 [11]. The following JH2 nondestructive constitutive equation of the Zr-based amorphous alloy material used in this study was obtained by fitting:

(1) $$\sigma_{i}^{*}=9.55{\left(p^{*}+0.43\right)}^{2.958}(1-0.035\ln\;\overset{\cdot }{\varepsilon }),$$

### 2.3. X-ray Test of Jet Formation

**Figure 5:**
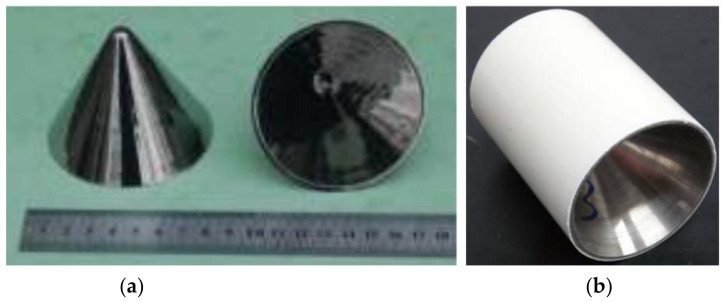
(**a**) Zr-based amorphous alloy liner and (**b**) the shaped charge.

The liner sample is shown in Figure 5a.

**Figure 6:**
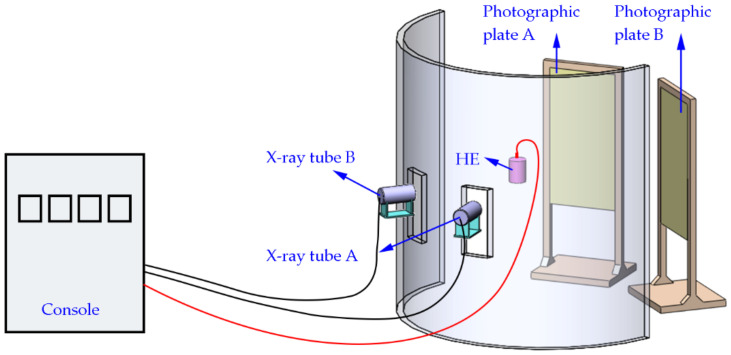
X-ray experimental configuration and setup.

**Figure 7:**
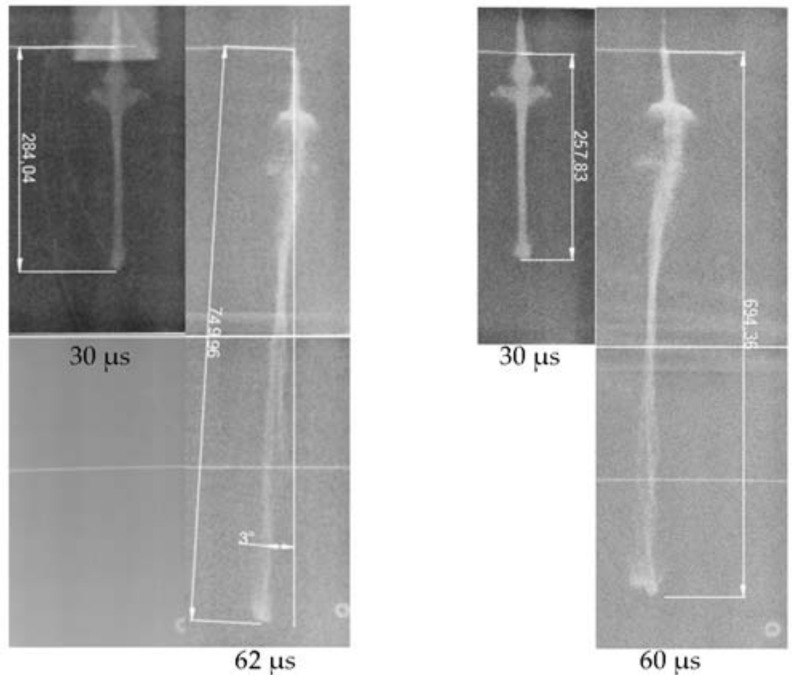
X-ray test result.

The site layout is shown in Figure 6, and the X-ray result is shown in Figure 7. The triggering time and the velocity of the jet tip (*v_j_*) are shown in Table 3.

### 2.4. Test of Zr-Based Amorphous Alloy Jet Penetrating Concrete

**Figure 8:**
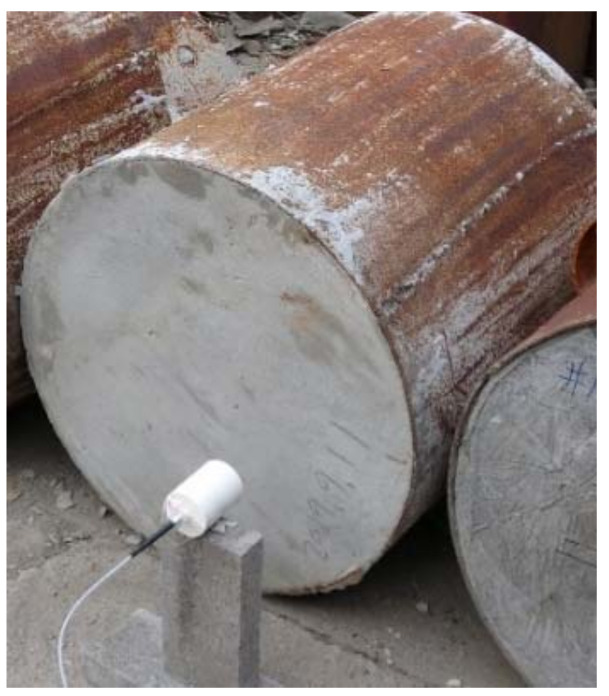
Depth-of-penetration (DOP) test site layout.

To further verify the high-speed particle flow form, hole formation, and penetration depth and to verify the high-speed particle flow penetration theory, a static DOP test of standard C35 concrete was conducted with a Zr-based amorphous alloy shaped charge based on the numerical simulation and theoretical analysis discussed above. The target plate was a cylindrical concrete pile with a density of 2.31 g/cm^3^, a thickness of 600 mm, and a diameter of 1000 mm. The cylindrical surface was surrounded by an iron sheet for restraint. Figure 8 shows the layout of the test site. The shaped charge was set 293 mm from the ground and 160 mm from the C35 concrete target plate, which was the same as the blasting height of the numerical simulation model mentioned above. The blasting method was #8 detonator center blasting.

## 3. Theory Study

### 3.1. Zr-Based Amorphous Alloy Incohesion Jet Formation Model

Under the action of a detonation wave, the Zr-based amorphous alloy shaped charge liner was crushed overall to form a large number of particles with varying speeds and masses, which converged axially along the liner busbar in the stagnation point coordinate system. After the particle flow collided in the axis stagnation point area, the particles were rearranged in space due to the influence of the uneven particle masses. The low-mass particles achieved higher radial velocities, forming a high-speed particle flow.

According to the analysis of the mechanical properties of Zr-based amorphous alloy material, it had low plasticity under dynamic conditions, whereas the elastic strain and yield strength were much higher than those of conventional metals. The elastic strain energy was high, and the plasticity was very low. The material underwent brittle cleavage under the high-strain-rate impact, and most of the elastic strain energy was converted into internal energy and released in the shear zone region.

For the convenience of calculation, the following assumptions were made. The Zr-based amorphous alloy liner material was assumed to be an ideal pure elastic solid. The elastic strain and corresponding elastic modulus before crushing were the dynamic response values under the impact strain rate and remained uniform throughout the liner. The elastic strain energy accumulated by impact loading during crushing was completely transformed into an adiabatic temperature increase and crack propagation, and it was dissipated in the form of internal energy. Other forms of dissipated energy during crushing were not included. The particle mass shapes obtained by crushing were the same, and the material porosity was 1, so the initial interface pressure could be simplified to a constant value (P_C-J_).

The chemical energy of the effective charge is equal to the sum of the kinetic energy of the gas after detonation, the kinetic energy of the particles, and the energy consumed in the particle fragmentation process:

(2) $$E_{explosive}{=E}_{gas}{+E}_{kinetic}{+E}_{loss},$$

**Figure 9:**
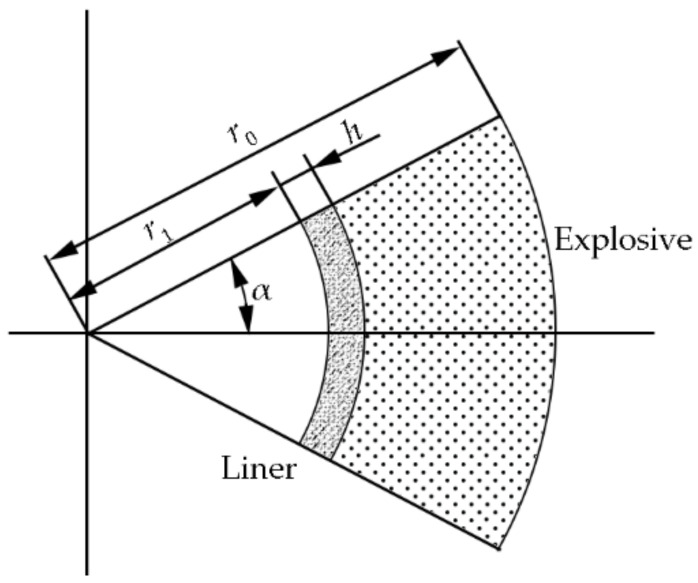
Control volume calculation diagram.

According to the momentum conservation law and the Gurney hypothesis, the liner can be regarded as a collection of a large number of microelements, as shown in Figure 9 and expressed as follows:

(3) $$\overset{r_{0}}{\underset{r_{1}}{\int }}\rho_{e}v_{g}rdr=\overset{\infty }{\underset{0}{\int }}\overset{r_{0}}{\underset{r_{1}}{\int }}{pdrd\;t+m}_{h}v_{0}.$$

where $\rho_{e}$ is the explosive density, $m_{h}$ is the unit mass of the liner, and $v_{g}$ is the final velocity of the gas after the detonation of the explosive. The velocity is assumed to be linearly distributed along the radial direction:

(4) $$v_{g}\left(r\right)=\left(v_{0}{+v}_{e}\right)\frac{r-r_{1}}{r_{0}-r_{1}}-{\;v}_{0}.$$

The energy conservation equation yields:

(5) $$m_{e}E-e_{loss}=\frac{1}{2}m_{h}v_{0}^{2\;}+\frac{1}{2}\overset{r_{0}}{\underset{r_{1}}{\int }}\rho_{e}v_{g}^{2}rdr.$$

According to the assumption that the maximum elastic strain energy of the material is approximately equal to the damage dissipated energy,

(6) $$e_{loss}=\frac{1}{2}\sigma \varepsilon sh,$$

where *σ* is the dynamic yield strength before fracture, *ε* is the dynamic elastic strain limit before fracture, *s* is the contact area of the liner and explosive under a unit mass, and *h* is the thickness of the liner.

Equations (3)–(6) can be combined to obtain the following:

(7) $$\begin{array}{l} v_{0}=\frac{-{A\;[8m}_{h}\left(r_{1}-r_{0}\right){+3\rho }_{e}-{5\rho }_{e}{\left(r_{1}-r_{0}\right)}^{2}]}{2\left(r_{1}-r_{0}\right){[\rho }_{e}{\left(r_{1}-r_{0}\right)}^{2}-{5m}_{h}\rho_{e}\left(r_{1}-r_{0}\right){+4m}_{h}^{2}]}\\ +\sqrt{\frac{A^{2}{{[8m}_{H}\left(r_{1}-r_{0}\right){+3\rho }_{e}-{5\rho }_{e}{\left(r_{1}-r_{0}\right)}^{2}]}^{2}}{4{\left(r_{1}-r_{0}\right)}^{2}{{[\rho }_{e}{\left(r_{1}-r_{0}\right)}^{2}-{5m}_{h}\rho_{e}\left(r_{1}-r_{0}\right){+4m}_{h}^{2}]}^{2}}-\frac{{2[6\rho }_{e}\left(m_{e}E-\frac{1}{2}\sigma \varepsilon sh\right)-{4A}^{2}\left(r_{1}-r_{0}\right)]}{\left(r_{1}-r_{0}\right){[\rho }_{e}{\left(r_{1}-r_{0}\right)}^{2}-{5m}_{h}\rho_{e}\left(r_{1}-r_{0}\right){+4m}_{h}^{2}]}}. \end{array}$$

*A* is the pressure distribution in the detonation gas:

(8) $${A=\mathrm{CP}}_{C-J}{\tau (r}_{0}-r_{1}{)(r}_{0}/r_{1}-1).$$

*m*_0_ is the mass per unit area of the liner, defined as $m_{0}{=\pi \rho }_{h}{(2r}_{1}h-h^{2})/r_{1}.$

The collapse velocity distribution is modeled via the Randers–Pehrson exponential formula:

(9) $$\begin{array}{c} v(t)=v_{0}[1-\exp(-\frac{t-t_{0}}{\tau })],\\ \tau =\frac{c_{1}{mv}_{0}}{P_{C-J}}{+c}_{2}. \end{array}$$

The projection angle is modeled via the Chou formula [12]:

(10) $$\delta =\frac{v_{0}}{2u}-\frac{\tau }{2}\frac{{dv}_{0}}{dl}+\frac{v_{0}}{4}\frac{d_{\tau }}{dl}.$$

The collapse angle is expressed as follows:

(11) $${\beta =\tan}^{-1}\frac{\sin\alpha -x\sin\alpha [1-\tan(\alpha +\delta ){\tan\delta ]v}_{0}^{\prime }/v_{0}}{\cos\alpha -2\sin\delta \sin(\delta +\alpha )+x\sin\alpha [\tan\left((\alpha +\delta \right){+\tan\delta ]v}_{0}^{\prime }/v_{0}}.$$

The particle flow velocity is calculated based on PER theory:

(12) $$v_{j}{=v}_{t}\csc\frac{\beta }{2}\cos(\alpha +\delta -\frac{\beta }{2}).$$

### 3.2. Penetration Model of Zr-Based Amorphous Alloy Incohesion Jet

Because the particle density of the particle flow was higher in the axial direction and the gap between particles was smaller with a low flying height, the particle flow could be considered to be approximately axially continuous. Owing to the expansion properties of the high-speed particle flow, both its diameter and length increased with time. The length and diameter of the particle flow element in the *i*th segment of the particle flow at stabilization time *t*_1_ are denoted as *l_jit_*_1_ and *d_jit_*_1_, respectively, and the length and diameter of the particle flow at the target time (*t_i_*) are:

(13) $$l_{jiti}{=l}_{jit1}{+\Delta v}_{ji}{(t}_{i\;}-{\;t}_{1}),$$

(14) $$d_{jiti}{=d}_{jit1}{+2v}_{jir}{(t}_{i\;}-{\;t}_{1}),$$

where ${\Delta v}_{ji}$ and *v_jir_* are the velocity difference between the head and tail of the *i*th section particle flow microelement and the radial velocity, respectively. The stabilization time (*t*_1_) and the radial velocity (*v_jir_*) after stabilization are constants, which were obtained by X-ray experiments.

The axial pores in the microelement of the high-speed particle flow can be regarded as the compressibility of the continuous jet. When the diameter of the particle flow was the smallest at stabilization time *t*_1_, the axial gap was the smallest and could be ignored. The target length was:

(15) $$l_{jit1real}{=l}_{jit1}\;(\frac{d_{jt1}}{d_{jiti}}{)}^{2},$$

Then, the penetration depth of the *i*th particle jet element is:

(16) $${\Delta P}_{i}{=u}_{i}{\Delta t}_{i}{=u}_{i}\frac{l_{jit1real}}{v_{ji}-u_{i}},$$

where ${\Delta t}_{i}$ is the penetration time of the *i*th segment microelement, and $u_{i}$ is the penetration speed of the *i*th segment particle flow microelement. $u_{i}$ is calculated by the axial penetration equation of the shaped jet:

(17) $$u_{i}=\frac{v_{ji}}{1+\sqrt{\frac{\rho_{t}}{\rho_{j}}}},$$

where $\rho_{t}$ and $\rho_{j}$ are target plate and jet density, respectively. The total penetration depth of the high-speed particle flow is:

(18) $$P_{n}=\overset{n}{\underset{i=1}{\sum }}{\Delta P}_{i}.$$

Analysis of the penetration process of the previous numerical simulation on the target plate revealed that when the particle density at the edge of the high-speed particle flow was less than a limit, its penetration effect on the target plate could be ignored. Therefore, the effective penetration diameter of the high-speed particle flow had an upper limit (*R_max_*), which was related to the strength of the target plate and the quality of the broken particles, which can be written as $R_{max}{(\sigma }_{t}{,m}_{f})$. Therefore, the small explosion height limit of the penetration model is:

(19) $$H\;\leq \;(\frac{R_{max}-d_{jit1}}{2v_{jir}}+t_{1}{)v}_{\mathrm{ji}\;}-\;h,$$

where *h* is the distance from the tip of the liner cone to the mouth.

Because the penetration behavior of the particle flow is regarded as the penetration behavior after being compressed into a condensed matter jet, the calculation of the radial aperture can be related to the condensed matter jet by the energy method:

(20) $$\frac{E_{\mathrm{ico}}}{V_{\mathrm{ico}}}=\frac{E_{\mathrm{inco}}}{V_{\mathrm{inco}}},$$

where $E_{i}$ and $V_{i}$ are the energy of the *i*th jet element at *t*_1_ and the volume of the penetration hole formed after penetrating the target plate, respectively. According to the assumption that the energies of the jet element and the penetration hole formed after penetrating the target plate are equal, the volume of the penetration hole is the same. The formula $V_{i}{=\pi D}_{i}^{2}P_{i}/4$ yields:

(21) $$D_{\mathrm{inco}}{=D}_{\mathrm{ico}}\sqrt{\frac{{\Delta P}_{\mathrm{ico}}}{{\Delta P}_{\mathrm{inco}}}}{=D}_{\mathrm{ico}}\sqrt{\frac{l_{\mathrm{jit}1\mathrm{co}}}{l_{\mathrm{jit}1\mathrm{nco}}}},$$

where $D_{i}$ represents the penetration aperture of the *i*th segment microelement, which can be obtained according to the theory of condensed jet penetration; $l_{\mathrm{jit}1\mathrm{nco}}$ is the length of the particle flow microelement during penetration; and $l_{\mathrm{jit}1\mathrm{nco}}{=l}_{\mathrm{jit}1\mathrm{real}}$. $l_{\mathrm{jit}1\mathrm{co}}$ is the length of the condensed jet microelement during penetration, which can be simply calculated as:

(22) $$l_{\mathrm{jit}1\mathrm{co}}{=l}_{\mathrm{jit}1}{+\Delta v}_{\mathrm{ji}}{(t}_{i}-t_{1}),$$

## 4. Numerical Simulation of Jet Formation

**Figure 10:**
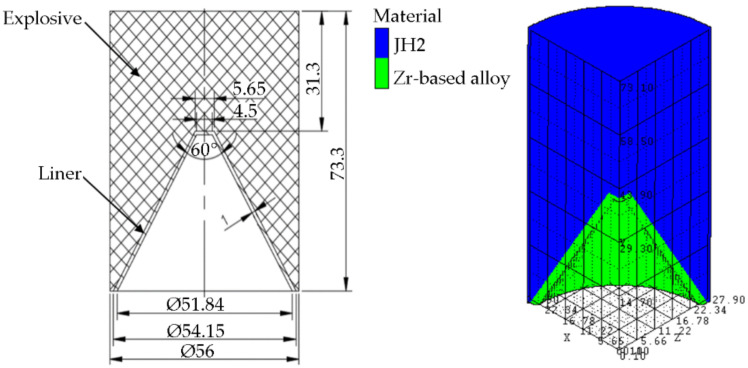
The charge structure and the simulation model.

A Ø56 mm standard shaped charge was modeled in this study. The charge had a height of 73.3 mm with a 1 mm thick Zr_41.2_Ti_13.8_Cu_12.5_Ni_10_Be_22.5_ cone liner and a JH2 explosive with a density of 1.72 g/cm^3^. The charge was detonated by a #8 detonator. The charge structure and the simulation model are shown in Figure 10. In the simulation, the smoothed particle hydrodynamics (SPH) model was used in Autodyn 3D in ANSYS 17.1(ANSYS, Canonsburg, PA, USA).

The Zr-based amorphous alloy was described by the Johnson–Holmquist strength model and failure model and a polynomial state equation. The parameters of the Zr-based amorphous alloy and the JH2 explosive are shown in Table 4 and Table 5, respectively.

**Figure 11:**
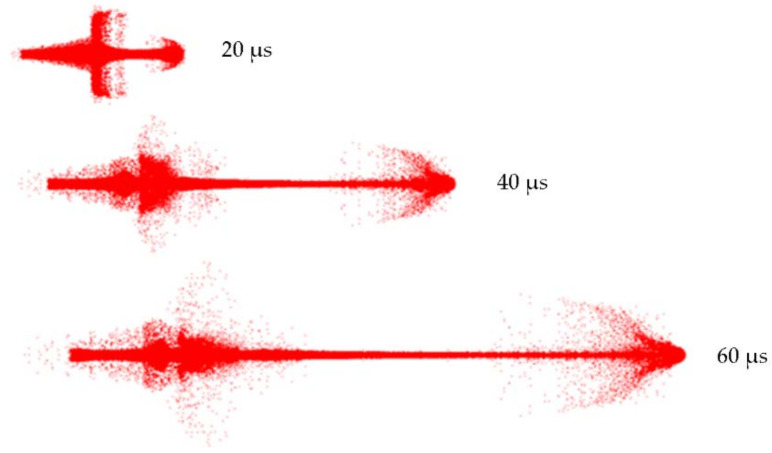
Zr-based amorphous alloy jet formation and stretching process.

The Zr-based amorphous alloy jet formation and stretching process are shown in Figure 11.

The jet tip velocity was 7252 m/s. At 60 µs, the continuity of the Zr-based jet was still good, and there was no necking phenomenon. The diameter of the Zr-based jet head increased continuously, showing incohesion characteristics. At 60 µs, the head diameter of the Zr-based jet reached 24.2 mm.

Chou et al. [12] studied the plane axisymmetric collision mechanism, and the flow formation criteria were determined as follows. When a subsonic collision occurred, a dense, condensed jet always formed. When a supersonic collision occurred, there was a maximum angle ($\beta_{c}$) for the formation of an attached shockwave. When the collapse angle (β) was greater than $\beta_{c}$, an incohesion jet formed, and when the collapse angle (β) was less than $\beta_{c}$, a jet was formed. The bulk sound velocity of the Zr-based amorphous alloy was 5824 m/s, far exceeding the crushing velocity of approximately 4000 m/s. Thus, the requirements of the sound velocity criterion for incohesion jets formed by traditional metal materials were not met. Therefore, the expansion characteristics of the Zr-based amorphous alloy jet heads were not affected by the sound velocity criterion, demonstrating that the jet properties of the Zr-based amorphous alloy differed from those of conventional metal jets.

**Figure 12:**
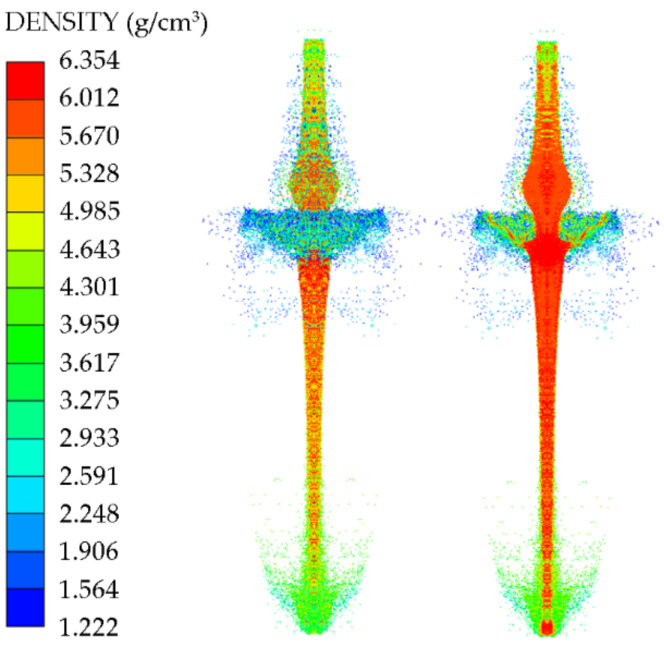
Cloud map of jet density distribution at 30 µs.

The particle density distribution at 30 µs is shown in Figure 12 based on the mass and density distribution of the Zr-based jet,. For the Zr-based jet, aside from the core part of the jet, the density of which was still above 5.6 g/cm^3^ at 30 µs, the outer density of the jet was generally low. The density was about 5.1 g/cm^3^, the density variation range was 91.7%, and the density distribution gradually increased from the external surface to the core. The Zr-based jet exhibited no necking phenomenon, the diameter of the main body of the jet remained basically unchanged, and the microelements of the head material were in a state of force equilibrium. The Zr-based jet was a high-speed particle flow.

A high-speed particle flow is a kind of jet that cannot form a condensed jet and slug due to the influence of the mechanical properties of the liner material during the collapse process of the liner, instead forming a high-speed particle beam state containing most of the mass. When the particle flow formed by the Zr-based jet collapse converged at the impact point, mass redistribution occurred after a large number of high-speed particles were impacted. Particles with a high density were less affected by radial forces, and the radial velocity was low inside the jet. The particles with a low density were significantly affected by the radial force and had a high radial velocity outside the jet. Furthermore, owing to the high brittleness of the material itself, some low-density particles were broken under heavy loading, which increased the density range.

## 5. Results and Discussion

### 5.1. Fracture Behavior of Zr-Based Amorphous Alloy

**Figure 13:**
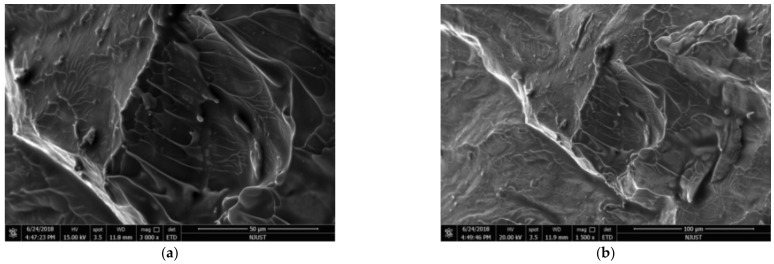
Dynamic fracture morphologies of Zr-based amorphous alloy specimens: (**a**) 3000 times magnification; (**b**) 1500 times magnification.

After experimentation, fragments of irregular size and shape were obtained. The tungsten carbide shims had partially melted small fragments that were peeled off and appeared together with the fragments recovered in the collection device. The fracture angles of the larger fragments were measured and found to be less than 45°. However, according to the Tresca criterion, the Zr-based amorphous alloy should be shear-fractured on the plane of maximum shear stress (that is, the shear fracture angle should be 45°). This difference was due to the extremely high fracture strengths of Zr-based amorphous alloys, which produced high normal stresses on the shear sections, which inhibited the development of shear fractures. This results in a shear fracture angle smaller than the maximum shear stress surface, indicating that the composition of the Zr-based amorphous alloy obeyed the Mohr–Coulomb criterion. The fracture surfaces of the specimens were observed by SEM, and the fracture morphologies are shown in Figure 13. Owing to the excessive number of fragments formed in the specimen, the local fracture morphology was selected to analyze the overall fracture morphology characteristics. Typical vein-like patterns were evident, but the Zr-based amorphous melting and droplet morphology were not evident near the vein pattern and in the crack propagation zone. The molten droplet was a result of the low thermal conductivity of the metallic glass and the high elastic strain. The elastic strain energy was high and released during local adiabatic shear, and the temperature in the shear band rose sharply to the glass phase-transition temperature or even close to the melting point temperature Tg of the material. The Tg of this Zr-based amorphous alloy was 932 K. The lack of molten droplets in the shear band may have been caused by the lack of structural defects of crystalline materials in the Zr-based amorphous alloy, the shear band not being fully developed during dynamic loading, the uneven elastic strain energy distribution, and the adiabatic shear fracture process first occurring in the local cluster structure with a partial stress concentration. Typical cleavage steps and accompanying river-like patterns are evident in Figure 13a,b, which are microscopic morphological characteristics that are unique to brittle cleavage fractures under dynamic shock. In the quasi-static tests, the loading rate was low, and the specimen required a long time to complete the shear compensation and the formation and propagation of secondary shear bands. In the dynamic test, the shear band did not form and develop sufficiently under dynamic conditions because the loading rate was much higher than that in the static state, and there was not sufficient time to complete the shear compensation, resulting in fewer secondary shear bands. This explains the experimental phenomenon of smaller fracture strains of the Zr-based amorphous alloy specimens under the room temperature dynamic tests than those under room-temperature quasi-static tests, as well as the more evident brittleness.

### 5.2. Results of X-ray Test, Theoretical Calculation, and Simulation of Jet Formation

**Figure 14:**
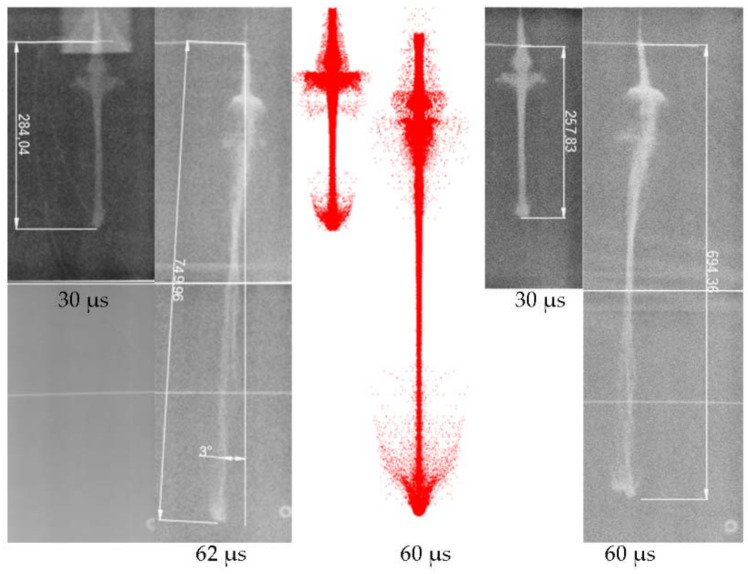
A comparison result of X-ray and numerical simulation of Zr-based jets.

According to the analysis presented Figure 14, 30 µs after initiation, the jet profile was clear, and the shape of each part of the jet can be clearly observed. The Zr-based jet exhibited satisfactory overall cohesiveness, with a slight expansion of the head and good continuity and symmetry of the whole jet. The image taken 60 µs after initiation shows that the overall continuity of the jet formed in the two tests was good, without necking, and the symmetry deteriorated. The overall edge of the jet was slightly fuzzy, resembling atomization, and the tail shape of the jet was significantly atomized. A comparison of the two time nodes showed that there was a transition section at the junction of the jet tail and the slug, with no evidence of morphological change. A collapse ring similar to the copper jet was not observed, but the particle swarm similar to the planetary belt was replaced because there was little effective charge at the bottom of the shaped charge liner, and the residual energy after crushing the liner was not sufficient to provide the particles with a high crushing speed. It was too late for the particles to converge to the axis, resulting in the bottom particle swarm flying around the high-speed particle flow. The above experimental phenomena are further proof that the jet existed in the form of a large number of high-speed particle flows, and a certain radial velocity was generated when the collision occurred, making the jet divergent.

A comparison of the pulsed X-ray photographs and the numerical simulation results shows that the appearances of the two were very close at the two times points, indicating that the SPH method could better describe the high-speed particle flow and the condensed jet and that the process was universal. Combined with the measurement data presented in Table 3 and Table 6, the simulated and measured values of the jet head velocity, head diameter, and jet length were in agreement, indicating the authenticity and reliability of the numerical simulation within this duration. For the Zr-based jet image, when *t* = 30 µs, the jet formation time was short, the radial travel of the edge particles was small, and the jet had not diverged significantly. When *t* = 60 µs, with the continuous movement of the particle flow, the low-density particles at the edge produced high displacement, and the atomization phenomenon was evident. In the numerical simulation, the main body of the jet showed no evident divergence, except at the head, possibly because the numerical simulation was more ideal than the experiment in calculating the process of the shaped charge liner crushing into particles. In the actual test, the particles crushed on the same circumference could not be guaranteed to have the same mass, causing variation in the radial velocities of particles, owing to differing momentum when the axis converged and collided, leading to the divergence of the main jet. Despite differences in the morphology, the relevant parameters of the Zr-based jet were still very close to the numerical simulation results. Thus, the numerical simulation can accurately simulate the formation performance of the jet within a certain range, and the data obtained from the numerical simulation can be used for analysis.

The theoretical calculation of the high-speed particle flow velocity formed by each microelement of the Zr-based amorphous alloy liner busbar under this working condition showed that the maximum velocity was 7298 m/s, which was 2.1% higher than the average particle flow velocity of the two tests. This was the result of only considering the elastic strain energy as the crushing energy, which reduced the total energy and increased the kinetic energy obtained by the particle flow. The theoretical model error was small, and it can be used to estimate the maximum velocity of the high-speed particle flow.

### 5.3. Result of DOP Test and Theorical Calculation of Jet Penetrate C35 Concrete

**Figure 15:**
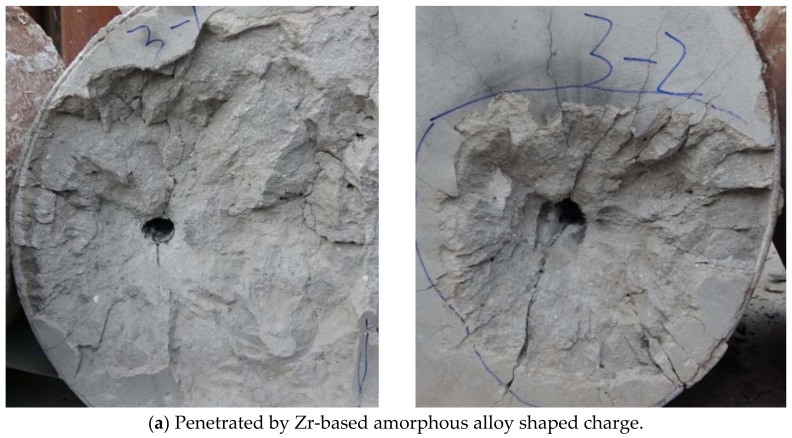

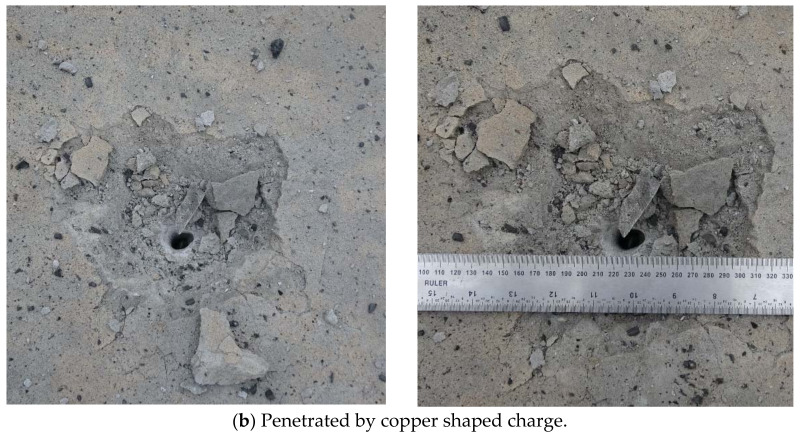
Penetration of shaped charge in two experiments: (**a**) penetrated by Zr-based amorphous alloy shaped charge; (**b**) penetrated by Cu shaped charge.

Two Zr-based amorphous alloy shaped charge DOP tests and one Cu shaped charge DOP test were conducted for comparison. The penetration and collapse of the shaped charge liner into the C35 concrete are shown in Figure 15, and the measured test data are shown in Table 7.

The crater diameter of the Cu shaped charge was about 180 mm (3.2 times the charging diameter), the depth of the funnel crater was 56 mm (equal to the charging diameter), the hole diameter (after the cleaning of the collapsed fragments) was about 25 mm (0.45 times the charging diameter), and the penetration depth (including the depth of the funnel crater) was 425 mm (7.59 times the charging diameter).

The average funnel crater diameter of the Zr-based amorphous alloy shaped charge was about 325 mm (5.8 times the charging diameter), the average depth of the funnel crater was 70 mm (1.25 times the charging diameter), the average hole diameter (after the cleaning of the collapsed fragments) was about 38 mm (0.68 times the charging diameter), and the average penetration depth (including the depth of the funnel crater) was 291.5 mm (5.2 times the charging diameter). Because the collapsed crater was deeper in the second test, the diameter of the entrance hole was slightly reduced. Moreover, the difference in the penetration depth was due to the concrete material characteristics, which caused the wall of the penetration hole to break and blocked the hole when it was penetrated. The penetration effects of the two tests were similar within a reasonable error range, indicating that the high-speed particle flow formed by the Zr-based amorphous alloy liner was relatively stable.

According to the above analysis, it can be concluded that although the penetration ability of the ZR-based shaped charge is less than that of the copper shaped charge (about 1.6 times that of copper), the ZR-based shaped charge achieves excellent performs in terms of hole-expanding ability, forming a large caving area and penetration hole size in the process of penetrating concrete.

A comparison of the theoretical and numerical simulation results with the experimental data are shown in Table 8.

A comparison of the theoretical model with the experimental data showed that, in addition to the collapse funnel crater (which is difficult to obtain by calculations owing to the influence of the concrete material properties, the unavoidable accumulation area at the bottom of the hole, and the penetration of the hole wall in the test), the blockage caused by crushing reduced the penetration depth smaller in the test measurement. The error of the theoretically calculated hole diameter of the collapse crater bottom was 5.56%, and the error of the penetration depth was 6.45%. The errors of the hole diameter and penetration depth at the bottom of the collapse crater were within a reasonable range.

## 6. Conclusions

In the present study, the particle flow crushing process of a Zr-based amorphous alloy was investigated based on the analysis of the mechanical properties of the alloy. The formed jet properties of the Zr-based amorphous alloy liner were obtained, the crushing process and formation characteristics of the high-speed particle flow were determined, and a calculation method for the high-speed particle flow velocity was proposed. Based on the penetration process of high-speed particle flow into concrete, the penetration stages were clearly distinguished, and a calculation method for target penetration was proposed. A static DOP test was used to verify the method, and a concrete penetration theory applicable to high-speed particle flow was obtained. The following findings were revealed:

(1) The Zr-based jet had good continuity. Because the Zr-based jet was composed of discrete particles, it did not shrink, owing to the velocity difference between the head and the tail. After the formation of the jet was stable, the density did not change with time, and the density distribution gradually decreased from the axis to the surface. Furthermore, a liner collapse model for a zirconium-based amorphous alloy liner was proposed.
(2) When the high-speed particle flow penetrated the target plate, it could be divided into transient impact, quasi-steady penetration, and penetration termination stages. The penetration depth of each stage accounted for about 10%, 85%, and 5% of the total penetration depth, respectively, and the reaming behavior occurred throughout the penetration process.
(3) According to the morphological characteristics of the high-speed particle flow, a calculation method for discontinuous jet penetration into target plate was proposed and verified through static DOP tests. A concrete penetration theory applicable to high-speed particle flow was obtained, providing a reference and basis for future applications of Zr-based amorphous alloy liners.

## Figures and Tables

**Table 1:** Zr-based amorphous alloy quasi-static compression test data.

Test	Constant Strain Rate (s^−1^)	Fracture Strain	Compressive Breaking Strength (MPa)
1	0.001	0.103	1400
2	0.001	0.092	1360

**Table 2:** Zr-based amorphous alloy test data.

No.	Constant Strain Rate (s^−1^)	Yield Strength (MPa)
1	2521	1135
2	2792	1282
3	3076	1162
4	3288	1028

**Table 3:** Triggering time and velocity of the jet tip.

Test	*t*_A_ (µs)	*t*_B_ (µs)	*v_j_* (m/s)
1	20.780	53.409	7231
2	20.864	51.914	7065

**Table 4:** Main parameters of the Zr-based amorphous alloy.

ρ (g/cm^3^)	Shear Modulus (GPa)	A	N	C	σ*_HEL_* (GPa)	*γ*
6.11	35.9	9.55	2.958	−0.035	6.15	1.85

**Table 5:** Main parameters of the JH2 explosive.

ρ (g/cm^3^)	A (GPa)	B (GPa)	R_1_	R_2_	ω	V_C-J_ (m/s)	P_C-J_ (GPa)
1.688	852	18.0	4.6	1.3	0.34	8300	29.6

**Table 6:** Comparison of numerical simulation and test results of jet formation.

Time (µs)	Measured Head Speed (m/s)	Simulated Head Speed (m/s)	Head Speed Error (%)	Actual Head Diameter (mm)	Dummy Head Diameter (mm)	Head Diameter Error (%)	Actual Jet Length (mm)	Simulated Jet Length (mm)	Jet Length Error (%)
62	7231	7252	0.3	23.6	25.2	6.7	369.6	356.2	−3.4
60	7065	7252	2.6	23.1	24.2	4.6	347.2	342.5	−1.4

**Table 7:** Test data of Zr-based amorphous alloy shaped charge penetrating C35 concrete.

No.	Material	Standoff (mm)	DOP (mm)	Enter Aperture (mm)	Collapse (mm) (Collapse Area × Collapse Depth)
1	Zr	160	273	Ø38	400 × 350 × 68
2	Zr	160	310	35 × 33	300 × 280 × 72
3	Cu	160		Ø25	200 × 160 × 56

**Table 8:** Comparison of theoretical model and experimental data.

	Inlet Hole Diameter (mm)	Aperture of Collapse Crater Bottom (mm)	Penetration Depth (mm)
Experimental results	-	Ø38	273
	-	35 × 33	310
Theoretical results	82	38	330

## Data Availability

Not applicable.
